# Case Report: A comprehensive case study on aggressive high-grade urothelial carcinoma of bladder that transforms into enteric-type adenocarcinoma along with an integrated treatment approach

**DOI:** 10.3389/fradi.2025.1586440

**Published:** 2025-06-09

**Authors:** Neha Rahul, Manishimwe Jules, Induni Nayodhara Weerarathna, Anurag Luharia, Prashik Dube

**Affiliations:** ^1^ Department of Radio Oncology, Datta Meghe Institute of Higher Education and Research (Deemed to be University), Wardha, India; ^2^ Department of Radiotherapy, School of Allied Health Sciences, Datta Meghe Institute of Higher Education and Research (Deemed to be University), Wardha, India; ^3^ Department of Biomedical Sciences, School of Allied Health Sciences, Datta Meghe Institute of Higher Education and Research (Deemed to be University), Wardha, India

**Keywords:** adenocarcinoma, urothelial carcinoma, chemotherapy, enteric-type adenocarcinoma, radiotherapy

## Abstract

Bladder cancer is a malignant tumour with a high morbidity and mortality rate in the world. Moreover, it is the most prevalent as well as commonly diagnosed in older individuals, with a median age of 73 years, and it has been reported that the most frequently seen histological type of bladder cancer was urothelial carcinoma. We present a unique case of a 44-year-old female with enteric-type adenocarcinoma, a rare and aggressive bladder cancer. Her symptoms included frequent micturition (Urination) and hematuria (blood in urine), at which point she was diagnosed with High-grade urothelial carcinoma. The malignancy worsened despite cycles of treatment requiring extensive surgery. After further tests, it was found that she had urothelial carcinoma with features of intestinal tissue (tissue of the intestine) and that the disease had infiltrated into nearby blood vessels and nerves. Radiation therapy was recommended to decrease the risk of local recurrence after surgery. The challenges in treating such a patient and the positive aspects this approach can give are highlighted in a case report.

## Introduction

Urothelial carcinoma, also known as transitional cell carcinoma (TCC), begins in urothelial cells, a type of cell that lines the inside of the bladder. These cells also line the inside of other urinary tract sections, including the ureters, the urethra, and the renal pelvis, the portion of the kidney that connects to the ureter (1). All urinary systems should be examined for tumours since people with bladder cancer occasionally have them elsewhere as well. High-grade tumours have a high malignant potential linked to notable progression and cancer mortality rates; bladder cancer is a diverse illness with a varying natural history (2).

Bladder cancer (BCa) is more common in males than in women. However, women have worse mortality rates than men. One of the reasons women have a greater death rate is that they are diagnosed with cancer at a later stage, making it more challenging to treat (3). Urothelial carcinoma typically affects elderly adults and is associated with chemical exposure as well as smoking. It is well acknowledged that high-grade variations with histological differentiation, such as squamous or glandular components, are aggressive and resistant to treatment. The lack of sufficient evidence needed to distinguish enteric-type adenocarcinoma from urothelial carcinoma makes therapy more difficult in the future (4). We report the case of a 44-year-old woman who acquired high-grade enteric and urothelial differentiation following major surgery, adjuvant radiotherapy, and several chemotherapy regimens.

## Case presentation

**Figure 1:**
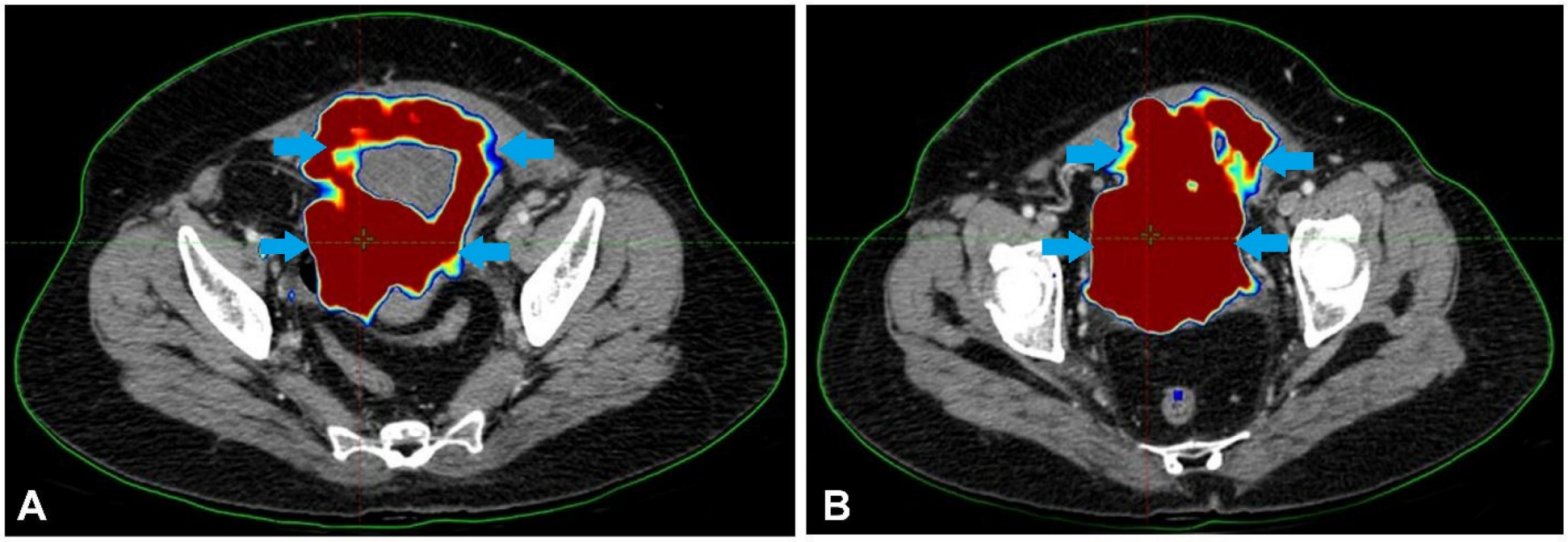
The initial PET-CT scan showed an irregular soft tissue mass at the bladder apex with areas of high FDG uptake, indicating active malignancy.

A 44-year-old lady with a history of haematuria and increased urine frequency for two and a half years presented with no other medical issues. Her maternal grandmother had previously had cervical cancer. Investigations and additional diagnostic testing were required since the first conservative treatment strategy was insufficient. These indicated an 8.1 × 7.4 × 5.7 cm soft tissue mass with calcified foci expanding irregularly toward the bladder's apex. Although the tumour did not spread to the neighbouring intestinal loops, it was detected near the uterine wall. A few small enhancing right external iliac and perivesical lymph nodes were seen. Figure 1 describes the initial PET-CT scan showing an irregular soft tissue mass at the bladder apex with areas of high FDG uptake, indicating active malignancy.

**Figure 2:**
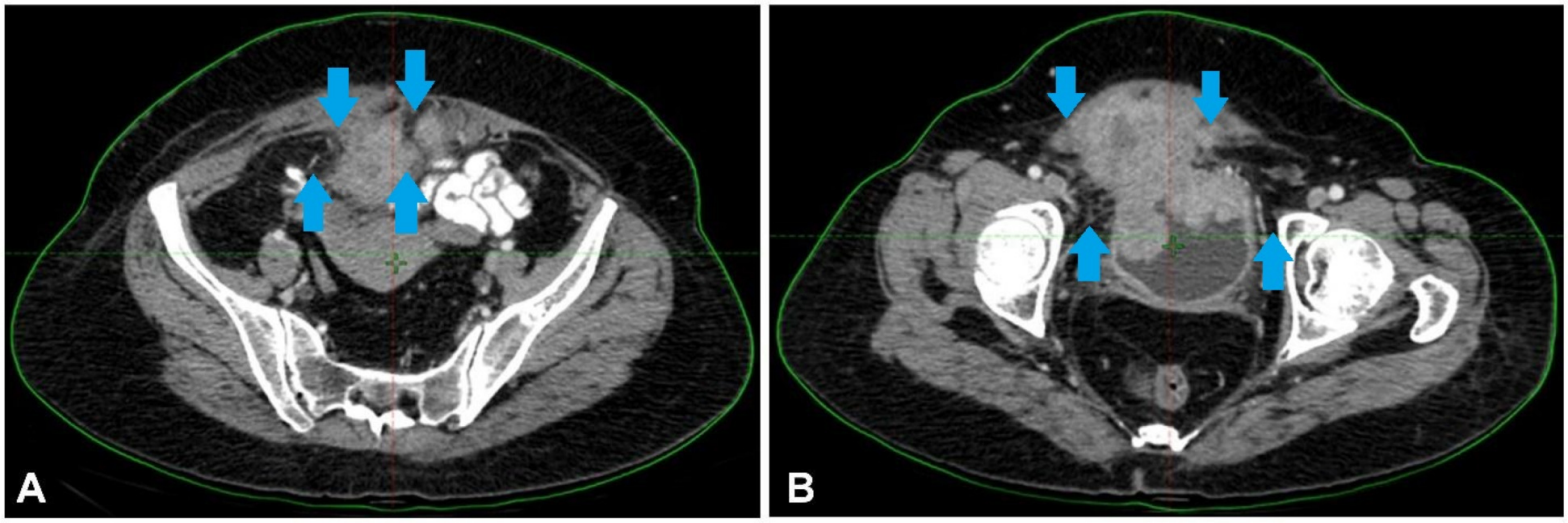
**(A**,**B)** Scan showing an irregular soft tissue mass at the bladder apex and asymmetric thickening of the bladder region.

The diagnosis of high-grade urothelial carcinoma with villoglandular features invading the lamina propria was validated by a pathology sample. The patient received four cycles of AUC2 Gemcitabine + Carboplatin therapy. However, the tumour persisted, leading to the therapeutic strategy reevaluation. A tumour that was highly active in FDG uptake, indicating significant invasion, was discovered by a PET-CT scan. Consequently, an MVAC treatment was used instead of the previous chemotherapeutic regimen. Despite three rounds of treatment, the cancer had progressed, as shown by PET-CT scans, and the tumour was 9.6 × 9.2 × 8.6 cm. Figure 2 shows the scan showing an irregular soft tissue mass at the bladder apex and asymmetric thickening of the bladder region.

**Figure 3:**
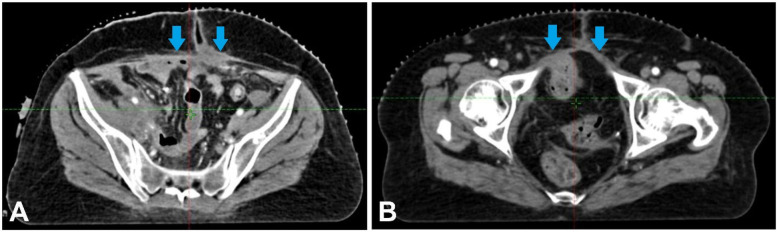
**(A**,**B)** Showing the post-surgery CT scan images with mild thickening involving the post-operative bed upto the scar site.

This suggests that radical anterior exenteration is the best surgical choice. The patient underwent ileal conduit construction, radical cystectomy, anterior exenteration, and complete abdominal hysterectomy with bilateral salpingo-oophorectomy. The resected mass's histological examination revealed the existence of enteric-type adenocarcinoma with invasion upto muscularis propria, Tumour size—9*10.8*8 cm., perineural and lymphovascular invasion and perivesical fat involvement. Area b/w bladder wall and uterine wall is positive. Nevertheless, there is no evidence of lymph node metastases out of the 30 lymph nodes dissected. Was seen. Endometrium, myometrium, cervix, bilateral ovaries, fallopian tubes- unremarkable. The patient was not affording for further testing of molecular markers, and pathological evaluation was not done after systemic therapy. Figure 3 depicts the post-surgery CT scan images with mild thickening involving the post-operative bed upto the scar site.

Consequently, adjuvant radiation was suggested to enhance local disease control. The extent of infiltration suggests **advanced disease progression**, necessitating comprehensive oncologic management. Table 1 summarizes the chronological sequence of clinical events, diagnostic findings, treatment interventions, and patient management outcomes.

**Table 1:** Timeline of diagnosis, treatment, and outcomes in the presented case.

Serial no.	Event	Timeline
1	Haematuria and increased urine frequency	For 2.5 years
2	8.1 × 7.4 × 5.7 cm soft tissue mass	Diagnosed with high-grade urothelial carcinoma
3	Chemotherapy with GemCarbo + AUC2 regimen	For 3 months
4	Response assessment	Stable disease with few aortocaval nodes
5	Received three cycles of chemotherapy with MVAC regimen	For 4 months
6	Response assessment	Progressive disease
7	Radical anterior exenteration with pelvic lymph node dissection	Within one month
8	Histopathology	MD Adenocarcinoma of the urinary bladder (Enteric type)
9	U/W Adjuvant Radiotherapy to post-op bed and + draining lymph node regions	In 1.5 months
10	On follow up	Completely disease free

## Discussion

A rare and severe kind of bladder cancer, highly differentiated urothelial carcinoma with enteric differentiation, was seen in this patient. The transformation of high-grade urothelial carcinoma (UC) to enteric-type adenocarcinoma is a rare but documented event, with limited data on the exact incidence and specific risk factors. While not fully understood, factors like pre-existing UC, chronic inflammation, and possibly specific genetic mutations may play a role. Adenocarcinomas of the bladder, including enteric-type, are a minority subset, with an incidence reported between 0.5% and 2.0% of all bladder carcinomas. The transformation of UC to adenocarcinoma is even rarer, and data on the specific incidence of enteric-type transformation is scarce.

**Figure 4:**
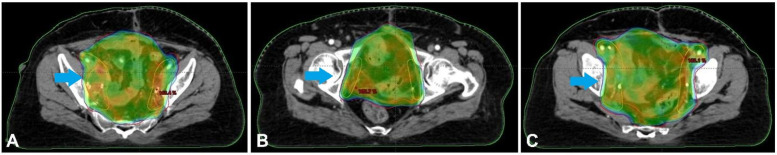
**(A**–**C)** The CT images display a radiotherapy treatment plan, with colour-coded dose distributions outlining the target volume and surrounding structures. The coverage indicates a high-dose region conforming to the tumour, ensuring optimal radiation delivery while sparing adjacent healthy tissues.

Because advanced bladder cancer spreads quickly, chemotherapy, the usual treatment for this kind of cancer, was unsuccessful (5). The patient's health deteriorated after many rounds of chemotherapy, prompting major bladder surgery (6). Since cancer had spread to the blood arteries and nerves bordering the tumour, radiation therapy was also recommended as adjuvant therapy to decrease the risk of recurrence (7). Radiation was administered to the post-operative bed, the draining lymph node regions, and adjacent areas at high risk of local recurrence. Figure 4 shows the **CT images** display a **radiotherapy treatment plan**, with colour-coded dose distributions outlining the target volume and surrounding structures. Figure 4 describes the **CT images** that display a **radiotherapy treatment plan**, with colour-coded dose distributions outlining the target volume and surrounding structures. The coverage indicates **a high-dose region conforming to the tumour**, ensuring optimal radiation delivery while sparing adjacent healthy tissues.

The patient has been cooperative with the treatment protocol with due consideration of her financial constraints. She understands the importance of regular and timely follow-up schedules and adheres to them. The patient is disease-free now and is in regular follow-up with necessary investigations at regular intervals. She has a functional and healthy urine stoma bag. Also, there were limitations in the treatment-related approach due to the lack of molecular profiling.

A diverse category of cancers with unique pathological subgroups and clinical manifestations is BCa. Roughly 90% of cases are classified as urothelial carcinoma (formerly known as transitional cell carcinoma), which is followed by squamous cell carcinoma and adenocarcinoma. Low- and high-grade types of urothelial carcinoma can occur, along with other histological variations, including villoglandular, micropapillary, and plasmacytoid characteristics (8).

Depending on the grade and stage of the tumour, different standard treatment approaches are used for BCa. The standard treatment for non-muscle-invasive bladder cancer (NMIBC) involves transurethral resection and intravesical therapy, such as Bacillus Calmette-Guérin (BCG). On the other hand, a more aggressive multimodal approach is usually needed for muscle-invasive bladder cancer (MIBC), which may need radical cystectomy after neoadjuvant chemotherapy or, in certain situations, bladder-preserving trimodal therapy. The paradigm for treating advanced and metastatic disease has also started to change due to recent developments in immunotherapy and molecular profiling (9).

Though it helps delay the recurrence of bladder cancer, especially when combined with other treatments, radiation therapy is now being studied for routine use for all types of bladder cancer. Specific subtypes of urothelial carcinoma (UC) are associated with poor outcomes; this patient highlights the necessity to personalize a treatment approach for patients with high-risk disease, and a multidisciplinary approach to care is required. This example illustrates the point that we require clinical research to understand better the role of radiation therapy both as a cytoreductive agent and as an additive therapy combined with chemotherapy and surgery for the management of aggressive variants of urothelial carcinoma.

Moreover, the case underscores the importance of individualizing the therapeutic approach based on the tumour's specific histological and genetic features. Because of intestinal differentiation with associated histological traits, including lymphovascular and perineural invasion, treatment has to be multidisciplinary. Oncologists, radiologists, pathologists and surgeons may work together to develop a complete treatment plan that accounts for the complexity of these extreme disease subgroups.

Moreover, the case reflects the difficulties of caring for a young child with a tumour somewhat atypical for his age. Although bladder cancer is most commonly seen in older patients, the potential for the disease to strike at a younger age, as seen here, means more work needs to be done to understand the genetic and environmental factors that may be involved in early-onset disease. Molecular analysis and comprehensive genetic counselling may reveal potential targeted therapies and help patients be better matched to personalized treatment plans.

Evolving evidence in Favor of successful high-grade urothelial carcinoma with intestinal differentiation management exposes the underlying need for an integrated battle plan that incorporates not only established but also forward-thinking treatment modalities.

## Conclusion

This case demonstrates the necessity of a multimodal treatment approach in the management of aggressive high-grade urothelial carcinoma with intestinal differentiation. Given the heterogeneity of this aggressive neoplasm characterized by an insatiable growth rate and resistance to conventional therapy, management needs to be both individualized and multifaceted. As enteric-type adenocarcinoma resulting from high-grade urothelial carcinoma is rare, more studies are necessary to understand its pathogenesis, identify biomarkers for early detection, and establish evidence-based recommendations for its treatment. This case also provides a multidisciplinary facet to a more holistic therapeutic approach, promoting teamwork between oncologists, radiologists, surgeons, and pathologists. Finally, better treatment options and knowledge regarding these rare and aggressive cancer subtypes could significantly improve patients’ survival and quality of life.

## Data Availability

The original contributions presented in the study are included in the article/Supplementary Material, further inquiries can be directed to the corresponding author.

## References

[1] Magi-Galluzzi C Falzarano SM Zhou M. Urothelial carcinoma and its variants. Surg Pathol Clin. (2008) 1:159–209. 10.1016/j.path.2008.07.004 26837906

[2] Klaassen Z Kamat AM Kassouf W Gontero P Villavicencio H Bellmunt J Treatment strategy for newly diagnosed T1 high-grade bladder urothelial carcinoma: new insights and updated recommendations. Eur Urol. (2018) 74:597–608. 10.1016/j.eururo.2018.06.024 30017405

[3] McKiernan J Asafu-Adjei D. Perspective: bridging the gender gap. Nature. (2017) 551:S39–S39. 10.1038/551S39a 29117165

[4] Nassar AH Umeton R Kim J Lundgren K Harshman L Allen EMV Mutational analysis of 472 urothelial carcinoma across grades and anatomic sites. Clin Cancer Res. (2019) 25:2458–70. 10.1158/1078-0432.CCR-18-3147 30593515

[5] Seidl C. Targets for therapy of bladder cancer. Semin Nucl Med. (2020) 50(2):162–70. 10.1053/j.semnuclmed.2020.02.006 32172801

[6] Calabrò F Sternberg CN. Neoadjuvant and adjuvant chemotherapy in muscle-invasive bladder cancer. Eur Urol. (2009) 55(2):348–58. 10.1016/j.eururo.2008.10.016 18977070

[7] Silina L Maksut F Bernard-Pierrot I Radvanyi F Créhange G Mégnin-Chanet F Review of experimental studies to improve radiotherapy response in bladder cancer: comments and perspectives. Cancers (Basel). (2020) 13(1):87. 10.3390/cancers13010087 33396795 PMC7795454

[8] Leslie SW Soon-Sutton TL Aeddula NR. Bladder cancer. In: StatPearls. Treasure Island, FL: StatPearls Publishing (2025). Available at: https://www.ncbi.nlm.nih.gov/books/NBK536923 (Accessed August 15, 2024). 30725608

[9] Zlotta AR Fleshner NE Jewett MA. The management of BCG failure in non-muscle-invasive bladder cancer: an update. Can Urol Assoc J. (2009) 3(6 Suppl 4):S199–205. 10.5489/cuaj.1196 20019985 PMC2792453

